# Oxide Ionic Neuro-Transistors for Bio-inspired Computing

**DOI:** 10.3390/nano14070584

**Published:** 2024-03-27

**Authors:** Yongli He, Yixin Zhu, Qing Wan

**Affiliations:** 1 Yongjiang Laboratory (Y-LAB), Ningbo 315202, China; yongli.he@ntu.edu.sg (Y.H.); yixin-zhu@ylab.ac.cn (Y.Z.); 2 National Laboratory of Solid-State Microstructures, Collaborative Innovation Center of Advanced Microstructures, School of Electronic Science and Engineering, Nanjing University, Nanjing 210093, China

**Keywords:** oxide semiconductors, ionic transistors, bio-inspired computing

## Abstract

Current computing systems rely on Boolean logic and von Neumann architecture, where computing cells are based on high-speed electron-conducting complementary metal-oxide-semiconductor (CMOS) transistors. In contrast, ions play an essential role in biological neural computing. Compared with CMOS units, the synapse/neuron computing speed is much lower, but the human brain performs much better in many tasks such as pattern recognition and decision-making. Recently, ionic dynamics in oxide electrolyte-gated transistors have attracted increasing attention in the field of neuromorphic computing, which is more similar to the computing modality in the biological brain. In this review article, we start with the introduction of some ionic processes in biological brain computing. Then, electrolyte-gated ionic transistors, especially oxide ionic transistors, are briefly introduced. Later, we review the state-of-the-art progress in oxide electrolyte-gated transistors for ionic neuromorphic computing including dynamic synaptic plasticity emulation, spatiotemporal information processing, and artificial sensory neuron function implementation. Finally, we will address the current challenges and offer recommendations along with potential research directions.

## 1. Introduction

The human brain efficiently processes information and interacts with the external environment, which relies on a neural network composed of one trillion neurons and one quadrillion synapses [1,2]. Computer engineers have long been intrigued by the remarkable energy efficiency of the biological brain when compared to cutting-edge silicon-based computing systems. A notable illustration of this contrast can be found in the case of the Bluegene supercomputer, which required megawatts of power to simulate the functioning of a cat’s brain. In contrast, the human brain only consumes ~20 W, encompassing cognition, control, movement, and decision-making concurrently [3]. Unlike digital circuits that depend on high-speed CMOS logic switches, the remarkable energy efficiency of the human brain’s intelligence is largely attributed to the dense synaptic interconnections among neurons [4]. Indeed, researchers are striving to achieve brain-like perception abilities with brain-like energy-efficiency through neuromorphic electronics [5]. Bionics offers a variety of inspirations like material design [6], bio-hybrid complex network design [7], and prosthetics [8]. By learning the computing paradigm of the human brain, neuromorphic computing aims to build computational hardware which mimics the biological nervous systems, and it is expected to play an essential role in the next era of high-efficiency hardware development [9].

Neuromorphic integrated circuits utilizing CMOS technology have been employed to replicate the cognitive and energy-efficient capabilities of the human brain. The most notable options include TrueNorth [10] and Loihi [11]. The CMOS technology is inherently inefficient for simulating synapses/neurons since it is not specifically designed for this purpose. Creating a functional synapse/neuron model in CMOS circuits requires multiple CMOS transistors. Neuromorphic devices, tailored specifically for simulating synapses/neurons, demonstrate enhanced efficiency when implementing synaptic and neuronal functions [12,13,14,15,16,17,18]. Neuromorphic device technologies include two-terminal memristive devices and multi-terminal transistor devices [19]. Two-terminal memristive device technologies comprise resistive random-access memory, phase-change memory, ferroelectric random-access memory, and magnetic random-access memory. Two-terminal memristive devices are characterized by a simple structure and the ability to realize extremely high integration density for cross-bar arrays, which can essentially speed up the most resource-intensive multiply–add operations in artificial neural networks (ANNs). However, two-terminal devices usually lack a selective terminal, which requires additional stackable two-terminal selectors or one-transistor one-memristor (1T1R) structures [17]. The multiply–add computation acceleration requires multibit non-volatile memory storage. Volatile memories possess the advantages of readily implementing the integrate-fire neuron model [20,21]. Three or multiple terminal transistor devices have a tradeoff between the integration density and other characteristics such as an improved control of the conductance state and a better emulation of neural functions [22]. Multi-terminal transistors, especially floating-gate transistors, have demonstrated advances in multiply–add operation accelerator circuits by virtue of multi-state storage and their compatibility with large-scale integrated circuits [23]. Compared with the resistive coupling of two-terminal memristors, the capacitive coupling of the transistor exhibits a lower static power and has a better resemblance with ionic capacitive information processing in biological neurons [22]. 

Biological neurons are primarily immersed in ion-rich body fluids. Neuronal capacitive coupling refers to the capacitance effect involving ionic charge accumulation, like Na^+^, K^+^, and Ca^2+^, on both sides of the neuron cell membrane. These ions play an essential role in generating an action potential (also called as a spike, a nerve impulse, or a discharge, about a few milliseconds width with an amplitude of 100 mV), maintaining the resting neuron membrane, and information transmission [24]. The neuron integrates the signal received from pre-synaptic neurons through synapses. When the integrated signal reaches the threshold of the neuron, it will fire an action potential and transmit the information to the post-synaptic neuron through the action potential. The action potential is the carrier of the information exchange between neurons. The synapse connects the neuron, and the connection strength can change as the signal flows through the synapse. This synaptic weight change is referred to as synaptic plasticity, which underlies the learning and memory functions of the biological neural network. The neuron encodes information into the frequency and pattern of action potentials to transfer information from one neuron to another. Indeed, the relatively lower ionic computing speed empowers the biological neural system’s excellent spatiotemporal information integration capabilities [25]. Unlike the CMOS electronic system that pursues the ultimate switching speed, biological synapses/neurons employ ions in body fluids to process information, which move much slower than electrons in silicon. Traditional CMOS transistors employ oxides like SiO_2_ or HfO_x_ as the gate dielectric capacitance to support the high-speed switching control of the channel. When the oxide dielectric is replaced with an ionic electrolyte, ionic transistors can be realized. The capacitance of the ionic transistor is formed by the anion/cation accumulation at the electrolyte/gate and electrolyte/channel interfaces. In principle, ionic transistors can simulate biological synaptic/neuronal functions more efficiently than CMOS transistors because the ionic transistor and biological neural networks both utilize ionic processes. Furthermore, the ionic dynamics provided by the electrolyte-gated transistors have the great potential for emulating spatiotemporal information processing similar to that of ionic information processing in biological nervous systems [26,27,28,29,30,31,32,33,34]. As early as 2010, a Si-based ionic transistor was proposed for synaptic ionic post-synaptic response emulation [26]. Recently, oxide semiconductors represented by indium-gallium-zinc-oxide (IGZO) have attracted worldwide research attention due to high mobility, large-area low-temperature preparation, and compatibility with microelectronics processes [35]. Wan’s group first proposed oxide semiconductor-based electrolyte-gated transistors for ionic synaptic/neuronal neuromorphic computation [28,36,37]. Subsequently, this domain experienced a surge in popularity, leading to the development of a wide variety of oxide ionic transistors for the emulation of ionic dynamic neural computation [38,39,40,41,42]. 

In this review article, we focus on recent advances of the oxide-based electrolyte-gated transistors for ionic neuromorphic computing. Biological synaptic/neuronal computing involving ionic processes is first introduced. Next, this article provides a brief introduction of the basic mechanism and outstanding advantages of the oxide-based ionic transistor. Then the latest progress in the field of oxide-based electrolyte-gated transistors for ionic neuromorphic computing is discussed. Finally, we give a short summary and outlook.

## 2. Ionic Processes in Brain Computing

**Figure 1:**
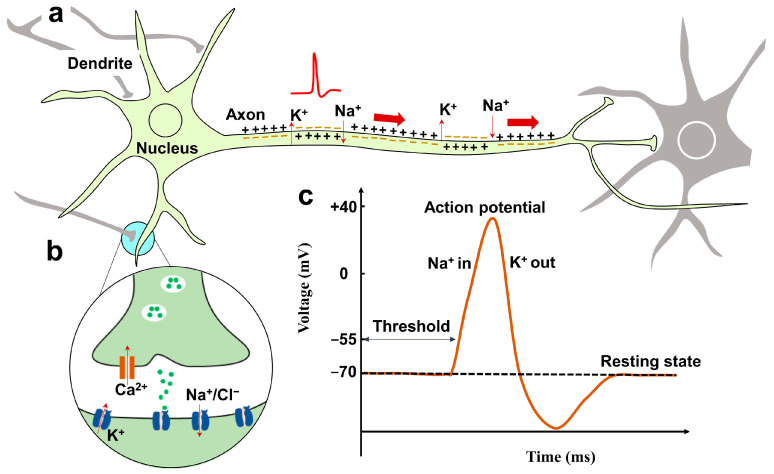
(**a**) Diagram of neuron, including dendrite, nucleus, and axon. (**b**) Enlarged synapse structure. (**c**) Schematic illustration of an action potential.

Neurons and synapses are the fundamental computing units of the human brain [43]. Neurons possess remarkable specialization in generating electrical signals upon receiving chemical and various stimuli, and then efficiently transmit these signals to neighboring cells. Figure 1a highlights some essential morphological specializations, including the dendrite responsible for receiving inputs from neighboring neurons and the axon responsible for transmitting the neuronal output to other cells. The axon forms synapses with the dendrite of other neurons through enlarged regions at their ends called pre-synaptic terminals. Axons transmit signals to neurons through the synapse. The cell transmitting signals is the pre-synaptic neuron, while the neuron receiving signals is the post-synaptic neuron.

Ions, especially Ca^2+^, Na^+^, K^+^, and Cl^−^, play a crucial role in neural signal generation, transmission, and processing [1]. As shown in Figure 1b, when the signal arrives at the axon terminal, it will stimulate the flow of Ca^2+^ into the pre-synaptic terminal. The influx of Ca^2+^ will stimulate the release of neurotransmitter, a type of endogenous chemical messenger that allows neurons to communicate with each other through synapses, to the synaptic cleft (usually with tens of nanometer width). Then the neurotransmitter diffuses through the cleft and binds with the receptor on the post-synaptic membrane. This binding will cause the opening of ion channels. If the opened channel is permeable to positive ions like Na^+^, as a consequence, the post-synaptic cell will experience depolarization, bringing it away from its resting membrane potential. Because it drives the membrane potential closer to the threshold required to generate the action potential, this effect is known as excitatory. This transient post-synaptic membrane depolarization is referred to as an excitatory post-synaptic potential (EPSP), and the corresponding current is an excitatory post-synaptic current (EPSC). If the opened channel is permeable to negative ions like Cl^−^, it will cause the hyperpolarization of the post-synaptic membrane, leading to an inhibitory post-synaptic potential/current (IPSP/IPSC). The synapse converts the neural signal from an electrical signal (action potential) into a chemical signal (neurotransmitter) and then converts it back to an electrical signal (post-synaptic potential). The above describes how chemical synapses transmit information. The chemical synaptic delay will be up to a few milliseconds, and the information transmission through the chemical synapse is unidirectional, that is, from pre-synaptic neuron to post-synaptic neuron. Other synapses are electrical, where the ions can flow directly between neurons. The information transmission of the electrical synapse is bidirectional. The electrical synapse cleft width is only a few nanometers, and the synaptic delay is less than 0.2 ms [44]. 

Normally, the neuron maintains a resting potential about −70 mV (potential difference between the inside and outside of a neuron), as shown in Figure 1c [45]. This negative potential is mainly maintained by unequal ion concentrations (like Na^+^ and K^+^) between the inside and outside of the neuron membrane. The concentration of these ions is regulated by the ion pumps which can transport ions across the cell membrane actively. When the post-synaptic potential reaches the threshold, a positive feedback process (Na^+^ entry) is initiated and it will emit an action potential. Then the K^+^ efflux will decrease the potential, and the neuron enters a refractory period (about 1 ms; during this period, it is impossible for the neuron to initiate another action potential). The action potential is transmitted along the axon to its terminal. Unlike the passively conducted electrical signals, action potentials are regenerated actively along the axon. Thus, action potentials travel across a long distance without attenuation. They maintain a consistent size and duration, making them reliable to transmit information. The increasing depolarization applied on the neuron has no effect on the neuronal output until it reaches the threshold; then an action potential fires. Therefore, the action potential is said to be ‘all-or-none’. This ‘all-or-none’ property makes it robust to noise, ensuring high resistance to interference. Our body receives the sensory information from the outside world, and sensory receptors code the external sensing information into spatiotemporal spike patterns. Not only which neuron the spike comes from carries information but also the timing between spikes. Information is transmitted between neurons via trains of action potentials.

## 3. Electrolyte-Gated Transistors

**Figure 2:**
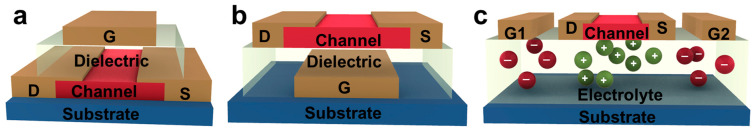
(**a**) Schematic structure of the top-gate transistor. (**b**) Schematic illustration of the bottom-gate transistor. (**c**) Schematic diagram of the lateral-coupling electric-double-layer transistor. G: gate, D: drain, S: source, G1: gate1, and G2: gate2.

The traditional thin-film transistor (TFT) has a sandwich structure consisting of the gate (G), dielectric, channel, source (S), and drain (D). As shown in Figure 2a,b, TFTs often use a top-gate or bottom-gate structure [46]. Taking n-type TFTs as an example, the identical source and drain areas are responsible for electrons’ provision and collection, respectively. The semiconductor region between the source and drain is referred to as the channel, allowing charge carriers (either electrons or holes) to move through it. The gate electrode has a low resistivity such as metal and covers the channel area. When gate voltage is applied, carriers accumulate at the channel/dielectric interface, modulating the conductivity of the channel. The dielectric layer, usually an oxide insulator, serves as a capacitor to regulate the carrier density at the channel/dielectric interface. The amount of carriers accumulated at the interface per unit area is the product of gate voltage (*V*_G_) and specific capacitance (*C*_S_). To reduce the operation voltage and energy consumption, higher specific capacitance is desirable. However, increasing of the capacitance of traditional oxide dielectrics results in reduced thickness. When the dielectric thickness is reduced below 10 nm, the increase of electron tunneling probability will greatly increase the leakage current, leading to the increase in static energy consumption [47]. 

**Figure 3:**
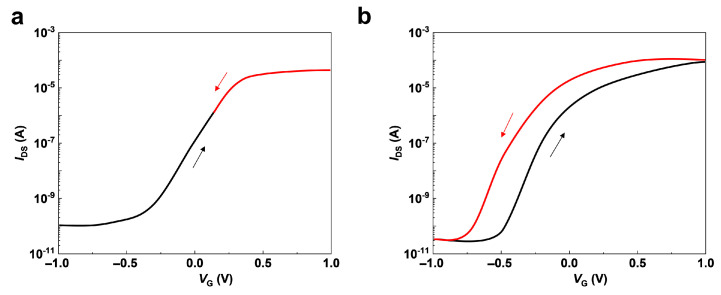
(**a**,**b**) Typical transfer curves of CMOS transistors and electrolyte-gated transistors when the gate voltage is scanned in the reverse direction, respectively.

The electrolyte can provide an extremely large specific capacitance through forming an electric-double-layer by ions’ accumulation at the gate/electrolyte and electrolyte/channel interfaces [48]. The electrolyte-gated transistor enables the implementation of lateral gate structures with gate-to-channel distances exceeding several centimeters, thanks to its remarkably high specific capacitance, as shown in Figure 2c [29]. It is difficult to envision in transistors with traditional oxide insulators. As we know, the transfer curve of CMOS transistor does not show hysteresis when the gate voltage is scanned in the reverse direction; that is, the two transfer curves of the forward and reverse scans overlap (Figure 3a). The transfer curve of ionic transistors typically shows a hysteresis when the gate voltage is scanned in the reverse direction (Figure 3b). The hysteresis occurs in the ionic transistor because the ions in the electrolyte move slowly. The relatively slow moving speed gives the electrolyte transistor tens of milliseconds to integrate the temporal signal. Recently, electrolyte-gated transistors have been extensively explored because much higher carrier densities can be realized compared to transistors with conventional oxide insulators. Oxide semiconductors [36], transition metal dichalcogenide-based semiconductors [49], organic semiconductors [50], carbon nanotubes [51], perovskite materials [52], etc., are explored as the channel material of electrolyte-gated transistors. Common electrolyte materials include polymer electrolytes [53], ionic liquids and gels [54], and inorganic electrolytes like microporous SiO_2_ [28], Al_2_O_3_ [55], zeolite [56], and Ta_2_O_5_ [57]. The electrolyte fabrication process includes spin-coating, plasma-enhanced chemical vapor deposition (PECVD), and sputter.

Oxide semiconductors represented by IGZO have attracted widespread attention in the field of panel display drive circuits due to their high mobility, low-temperature preparation over large-areas, and high device consistency [35,58]. In 2003, H. Hosono et al. invented an oxide transistor with a crystal IGZO channel, and the carrier mobility exceeds ~80 cm^2^V^−1^s^−1^ [59]. One year later, the same group reported a flexible transparent oxide transistor with an amorous IGZO channel, and the carrier mobility exceeds ~10 cm^2^V^−1^s^−1^ [35]. Compared with a-Si, there has been a significant improvement in mobility from ~1 to ~10 cm^2^V^−1^s^−1^. The bandgap width of the IGZO semiconductor is about 3.0 eV. The wider bandwidth of the IGZO semiconductor compared with silicon (bandgap of about 1.1 eV) offers various applications for the IGZO transistor, such as transparent electronics and low-off current. The channel length scalability of the oxide transistor is proved to be promising down to 5 nm [60]. The fabrication methods of the IGZO semiconductor include sputtering, atomic layer deposition, pulsed laser deposition, electrospinning, and spin-coating. These low-temperature thin-film deposition methods make oxide semiconductors promising for the application of flexible electronics and also makes oxide transistors compatible with most electrolyte processing processes, which are typically organic materials.

In 2009, Wan’s group reported the first IGZO ionic transistor with inorganic SiO_2_ electrolyte [36]. Then they developed a variety of oxide ionic transistors, in which the channel materials include IGZO [61,62,63], IZO (indium–zinc–oxide) [64], ITO (indium–tin–oxide) [65], IWO (indium–tungsten–oxide) [66] and the electrolyte materials include microporous SiO_2_ [36], chitosan [32], etc. In 2013, they pioneered the development of the first flexible neuromorphic devices using oxide ionic transistors [37]. Moreover, they have since advanced their research to create versatile neuromorphic devices based on oxide ionic transistors. This field has attracted widespread attention as more and more researchers are interested in successfully implementing some essential ionic neural functions such as synaptic plasticity [67,68,69,70,71,72,73,74,75,76,77,78,79,80,81,82,83,84,85], synaptic filtering [86,87,88], synaptic learning rules [89,90,91,92,93,94], neuronal coding [95], neuronal integration [96], spatiotemporal information processing [32,97], reservoir computing [63], artificial neural networks [41,74,98,99,100,101,102,103,104,105,106], and artificial sensory neurons [107,108,109,110,111,112,113,114]. Subsequent chapters describe recent advances in this field.

## 4. Dynamic Synaptic Plasticity in Oxide Ionic Transistors

Synaptic plasticity is believed to be the foundation of information processing, memory, and learning abilities [115]. The implementation of synaptic plasticity plays an essential role in realizing ionic dynamic neuromorphic computing. A wide variety of synaptic ionic computing behaviors such as post-synaptic currents [41,100,116,117,118,119,120,121,122,123,124], short-term plasticity [29,64,69,125,126,127,128,129,130,131,132,133,134,135,136,137], long-term plasticity [138,139,140,141], and synaptic learning rules [142,143,144] have been implemented by oxide ionic transistors.

### 4.1. Short-Term Plasticity

**Figure 4:**
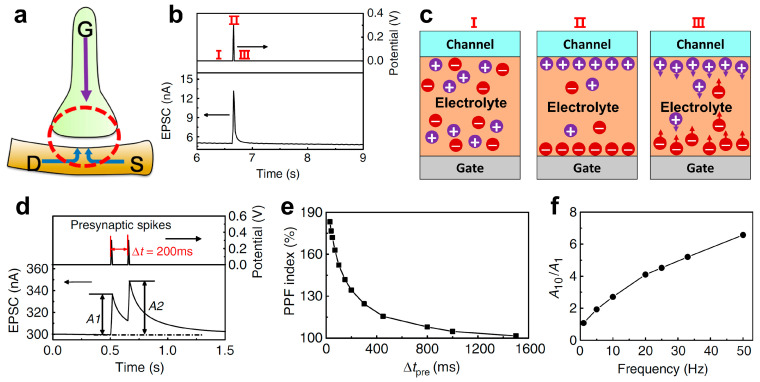
(**a**) Schematic illustration of an artificial synapse based on an oxide ionic transistor, where the gate is used as the pre-synaptic terminal and source/drain is used as the post-synaptic terminal. (**b**) Post-synaptic current (*I*_DS_) triggered by a pre-synaptic pulse (electrical gate pulse). It shows three stages of the pre-synaptic voltage pulse (before, when, and after pulses). (**c**) Ion distributions in the gate electrolytes under three stages (I: before, II: when, and III: after the electrical gate pulse). (**d**) Paired-pulse facilitation triggered by two successive electrical gate pulses. (**e**) Paired-pulse facilitation index as a function of the time interval between the two pre-synaptic pulses. (**f**) High-pass filter gain as a function of pre-synaptic pulse frequency. (**a**) is reproduced from ref. [145]. Copyright 2016 American Chemical Society. (**b**,**d**–**f**) are reproduced from ref. [28]. Copyright 2014 Springer Nature.

Using the gate as the pre-synaptic terminal and the source-drain as the post-synaptic terminal, ionic synaptic function can be simulated in oxide ionic transistors, as shown in Figure 4a [145]. To emulate neural dynamic functions, let us consider the simplest case—the emulation of EPSC triggered by a single pulse [28]. When a voltage pulse is applied on the gate terminal, the ions with opposite polarities in the electrolyte will move from their equilibrium positions to the gate/electrolyte interface and the electrolyte/channel interface, resulting in an increase in the channel current (Figure 4b and Figure 4c-II). After the gate pulse’s removal, the ions at the interfaces will take a few tens of milliseconds to diffuse back to their equilibrium positions (Figure 4c-III). Therefore, the current in the channel also slowly decays back to its basement value, mimicking biological EPSC characteristics [1]. 

The diffusion features of the EPSC play an essential part in time-dependent information processing in synapses [146]. Paired-pulse facilitation (PPF) is a type of short-term plasticity which is responsible for processing continuous pre-synaptic pulses with a short time interval [147]. It describes that the post-synaptic current triggered by the second pre-synaptic stimulus (A2) is larger than that triggered by the first one (A1), and the PPF ratio (A2/A1) decreases with the time interval increases. As shown in Figure 4d, when two successive electrical pulses are applied on the gate terminal, the PPF function is mimicked. When the second pulse arrives, some ions accumulated at the electrolyte/channel interface triggered by the first pulse have not diffused back to their equilibrium positions. These remaining ions will overlay with the ions triggered by the second electrical pulse. This will result in an increase in the response, emulating the PPF function. The shorter the time interval is, the more residual ions will remain at the interface and the larger the PPF ratio is (Figure 4e). The synaptic information processing relies heavily on the pre-synaptic patterns including the number and the frequency of the pulses. As the PPF ratio decreases with increasing time intervals, high-pass filtering can be realized which means that higher-frequency gate pulses result in larger channel current response (Figure 4f).

### 4.2. Long-Term Plasticity

Ionic diffusion processes last only tens of milliseconds to seconds, which is suitable for emulating short-term plasticity. [26] However, long-term synaptic plasticity that lasts for a long period or even a lifetime is the basis for memory and leaning abilities [148], which cannot be realized by ionic diffusion processes. Some mechanisms like electrochemical doping [37,64,145,149,150,151,152,153,154,155], chemical reaction [156], Schottky barrier height modulation [61,62,157], and phase-change [158,159] are developed to modulate the conductivity of the channel, emulating the long-term synaptic plasticity in oxide ionic transistors.

**Figure 5:**
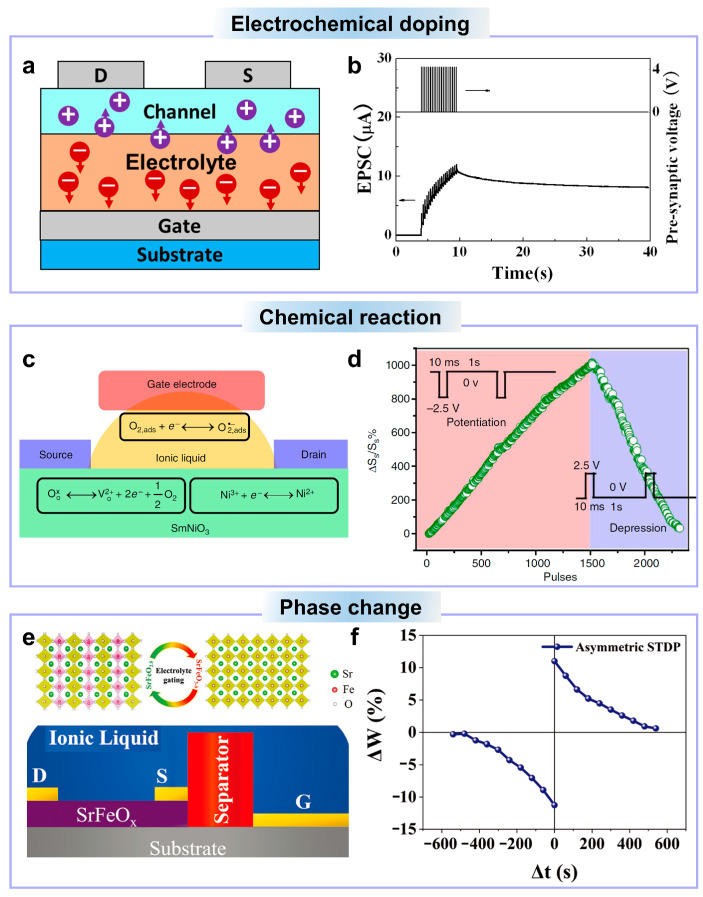
(**a**) Schematic illustration of ionic electrochemical doping of the oxide channel. (**b**) Post-synaptic current triggered by high positive gate electrical pulses. (**c**) Chemical reaction-based channel conductance modulation mechanism of SmNiO_3_-based ionic transistors. (**d**) Channel conductance modulation of the SmNiO_3_-based ionic transistor under positive/negative gate pulses. (**e**) Phase change-based channel conductance modulation mechanism of the SrFeO_x_-based ionic transistor. (**f**) Emulation of STDP in the SrFeO_x_-based ionic transistor. (**a**,**b**) are reproduced from ref. [37]. Copyright 2013 IEEE. (**c**,**d**) are reproduced from ref. [156]. Copyright 2013, Springer Nature Limited. (**e**,**f**) are reproduced from ref. [159]. Copyright 2019 WILEY-VCH Verlag GmbH & Co. KGaA, Weinheim.

Semiconductor doping can greatly modulate the conductivity of the channel. As early as 2013, Zhou et al. proposed a flexible IZO ionic synaptic transistor for long-term synaptic plasticity emulation by electrochemical doping [37]. It is reported that hydrogen is a type of donor doping of zinc oxide [160,161]. As shown in Figure 5a, when a sufficiently high positive electrical pulse is applied on the gate terminal, some hydrogen ions in the electrolyte will penetrate into the channel to electrochemically dope the IZO channel. After doping, channel conductance is greatly enhanced, as shown in Figure 5b. This conductivity enhancement can last for a long time, achieving the emulation of long-term synaptic plasticity. However, it can only realize the long-term potentiation plasticity because of the permanent doping of the channel, which is a non-reversible process.

The redox reaction can reversibly change the conductivity of the oxide channel, which can be used for the emulation of both long-term potentiation and depression plasticity. Shi et al. proposed a type of SmNiO_x_ electrochemical synaptic transistor for long-term synaptic plasticity emulation [36]. The ionic liquid is used as the electrolyte, and SmNiO_x_ is used as the oxide channel. As shown in Figure 5c, the operating principle of the electrochemical device can be succinctly explained as follows: when a positive gate voltage is applied, oxygen exits the SmNiO_x_ material by creating oxygen vacancies, resulting in the reduction of Ni^3+^ to Ni^2+^. Conversely, under negative gate pulses, oxygen, whether initially stored in the ionic liquid in gaseous form or regenerated from superoxide, becomes part of the SmNiO_x_ lattice, facilitating the oxidation of Ni^2+^ back to Ni^3+^. This reversible reaction realizes the reversible modulation of the SmNiO_x_ conduction, making it possible to emulate both the long-term depression and potentiation plasticity. Figure 5d shows reversible channel conductance caused by negative/positive gate pulses, which emulates the long-term potentiation and depression plasticity. In addition, it realized more than 900 conductance states, mimicking multiple synaptic weights.

Phase change is an essential mechanism for conductance modulation, and phase-change memory relies on the transformation of crystalline and amorphous states of phase-change material [162]. Ge et al. used the phase-change mechanism in oxide ionic synaptic transistors for long-term plasticity and synaptic learning rule implementation [159]. Ionic liquid and SrFeO_x_ are used as the electrolyte and the channel, respectively. As shown in Figure 5e, the control of topotactic phase transformation between brownmillerite SrFeO_2.5_ and perovskite SrFeO_3−δ_ by electrolyte-gating is used to emulate multiple synaptic levels. As one of the synaptic learning rules, the spike-timing dependent plasticity (STDP) describes the dependence of the synaptic weight change on the time difference between pre- and post-synaptic spikes [163]. To mimic the STDP learning rules, a time difference between the electrical pulses is applied on the pre- and post-synaptic terminal (gate and source/drain). As shown in Figure 5f, the percentage of conductance change with different polarity time difference suggests the emulation of STDP functions. However, the time difference between pre- and post-synaptic terminals is enlarged to several hundreds of seconds in the emulation, which is several tens of milliseconds in the biological synapse. This is limited by the low working speed of the electrolyte-gated device, which can be solved by employing electrolytes with fast ion response.

## 5. Spatiotemporal Information Processing in Oxide Ionic Transistors

Sensory organs like eyes, ears, and noses receive spatiotemporal information from the environment, which plays an essential part for predation, danger avoidance, courtship, etc. The information transmission from pre-synaptic neurons to the dendrites of post-synaptic neurons occurs at synapses, and information is encoded by the precise timing of synaptic spikes (temporal) originating from different pre-synaptic terminals (spatial) [164,165,166]. This spatiotemporal coding mechanism in biological neural systems empowers the human brain to efficiently represent dense information because both spatial and temporal factors convey crucial data. The ability to distinguish diverse spatiotemporal input sequences stands as a fundamental necessity in the processing of sensory information. Therefore, implementation of spatiotemporal information processing is very important for ionic dynamic neuromorphic computing.

**Figure 6:**
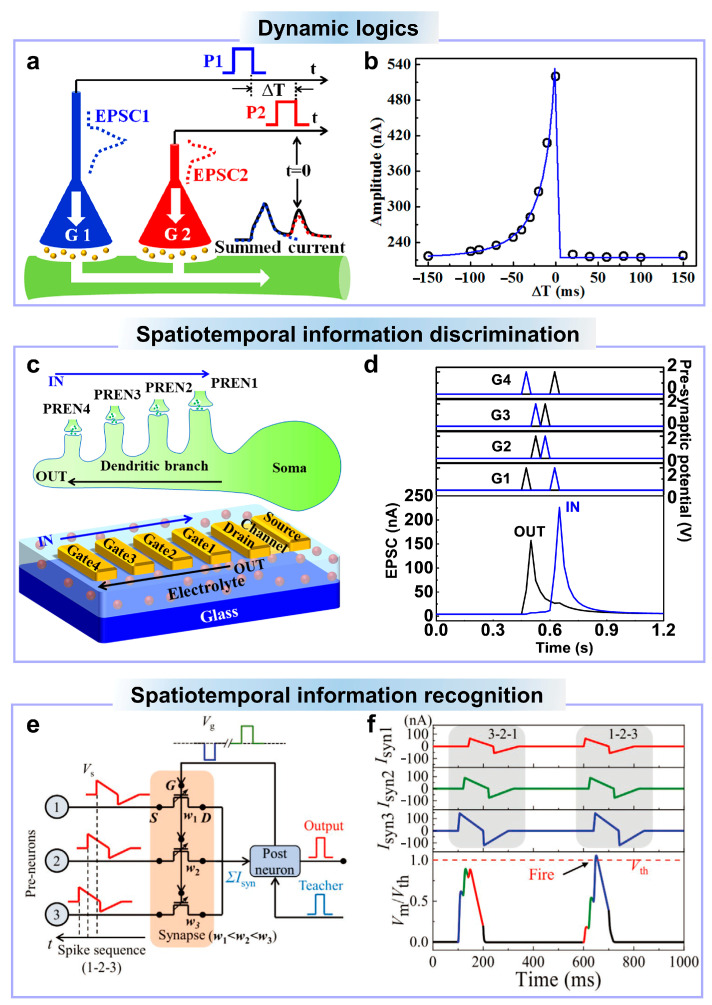
(**a**) Schematic illustration of the gate pulse pattern applied on the oxide ionic transistor for dynamic logic emulation. (**b**) Post-synaptic current amplitude plotted as a function of the time interval between each pulse. (**c**) Schematic structure of a single synaptic dendrite and a schematic sturcture of an IGZO ionic transistor with multiple gates in one direction, mimicking pre-synaptic terminals on a single dendritic branch of a neuron. (**d**) Post-synaptic response to activation pulse sequences in the IN and OUT direction. (**e**) Schematic diagram of a 3 × 1 SNN with three pre-synaptic neurons connected to one post-synaptic neuron through three oxide ionic transistors. (**f**) Measured post-synaptic current through each synapse and the simulated membrane potential with different pulse sequences like 3–2–1 and 1–2–3. (**a**,**b**) are reproduced from ref. [169]. Copyright 2016 AIP Publishing LLC. (**c**,**d**) are reproduced from ref. [32]. Copyright 2019 WILEY-VCH Verlag GmbH & Co. KGaA, Weinheim. (**e**,**f**) are reproduced from ref. [97]. Copyright 2020 Wiley-VCH GmbH.

The lateral strong ionic/electronic coupling effect of the electrolyte-gated transistor makes it possible for multiple gate input terminals [167]. Ionic temporal coupling and multi-gate spatial coupling make the electrolyte-gated oxide transistors feasible for biological spatiotemporal information processing [32,168]. Wan et al. proposed a multiple lateral gate ionic oxide transistor for dynamic logics by applying spatiotemporal correlated spikes [169]. An ionic methylcellulose film is used as the electrolyte, and an IZO oxide semiconductor is used as the channel. Double lateral gates are used as the pre-synaptic terminals, as shown in Figure 6a. There is a time difference between the first pulse applied on gate1 and the second pulse applied on gate2. Zero time is defined as the time when the pulse applied on gate2 ends. As shown in Figure 6b, if the time difference is smaller than 0, the pulse applied on gate1 leads the pulse applied on gate2. The post-synaptic response caused by the pulse applied on gate1 will be superimposed with that triggered by the pulse applied on gate2 due to ion-electron coupling. The smaller the time interval, the more residual ions will be augmented with that triggered by the pulse applied on gate2, and the larger the response will be. If the time difference is larger than 0, the pulse applied on gate1 will lag behind the pulse applied on gate2. The measured response is only that caused by the pulse applied on gate2.

The discrimination of spatiotemporal spike sequences from pre-synaptic terminals by dendrites is believed to be the basis of spatiotemporal information processing [170]. He et al. proposed capacitive-coupled multi-terminal IGZO ionic neuro-transistors for dynamic spatiotemporal information processing, simulating the discrimination of diverse spatiotemporal input sequences akin to dendritic processes [32]. By utilizing a substantial electric-double-layer capacitance, these capacitive-coupled multi-terminal neuro-transistors exhibit remarkably low power consumption, effectively replicating the dendritic discrimination of diverse spatiotemporal input sequences by applying spatiotemporal electrical pulse sequences on the gate electrodes. As shown in Figure 6c, in order to replicate the differentiation between various sequential activations of a specified group of synapses on a single dendritic branch, spatiotemporal electrical pulse sequences are employed on the multiple gate electrodes in a specific direction of the neuro-transistor, each with distinct gate-to-channel distances. As shown in Figure 6d, we can clearly see that the IN direction pulse activation (from the dendrite branch to the soma) produces a larger response than the OUT direction (from the soma to the tip). For the IN direction, electrical pulse sequences are applied to the gate electrodes sequentially, starting with the one farthest to the channel, resulting in a decreasing order of the distance. As a result, the relaxation time for protons at the channel/electrolyte interface to return to their equilibrium position also decreases. Consequently, the residual protons become more closely coupled to those triggered by subsequent electrical pulses due to the extended diffusion time. Thus, sequential activation of the neuro-transistor in the IN direction leads to the larger response peak. This demonstrates the ability to recognize spatiotemporal sequences.

In 2020, Li et al. proposed a Nb_2_O_5_ ionic transistor for spatiotemporal information processing [97]. In order to realize the recognition, a straightforward 3 × 1 spiking neural network (SNN) was established. As illustrated in Figure 6e, three pre-synaptic neurons are connected to one post-neuron through three oxide ionic synapses. Furthermore, the synaptic weight values were intentionally arranged in the ascending order, namely *w*_1_ < *w*_2_ < *w*_3_. Figure 6f displays the recorded post-synaptic current (*I*_syn_) and the computed membrane potential (*V*_m_) when inputting two three-spike sequences with timing conditions: t_1_ < t_2_ < t_3_ and t_1_ > t_2_ > t_3_, referred to as “1–2–3” and “3–2–1”, respectively. Notably, a higher membrane potential (*V*_m_) is observed for the “1–2–3” spike sequence due to the positive weight-time correlation. This device demonstrates the ability to emulate spatiotemporal information processing.

## 6. Artificial Sensory Neurons by Oxide Ionic Transistors

The sensory system serves as the crucial bridge between the external physical world and human perception, facilitating the transmission and processing of environmental stimuli to enable us to comprehensively understand and consciously experience our surroundings [1,24]. Through the integration of sensors with bioinspired cutting-edge synaptic/neuronal devices, researchers have successfully engineered artificial sensory systems that closely mimic the intricate functions of their biological counterparts [110,171]. These innovative artificial sensory systems not only replicate the sensory information processing mechanisms found in nature but also offer the potential to revolutionize various fields, such as robotics, healthcare, and human–computer interaction, by enhancing sensory perception and adaptability.

**Figure 7:**
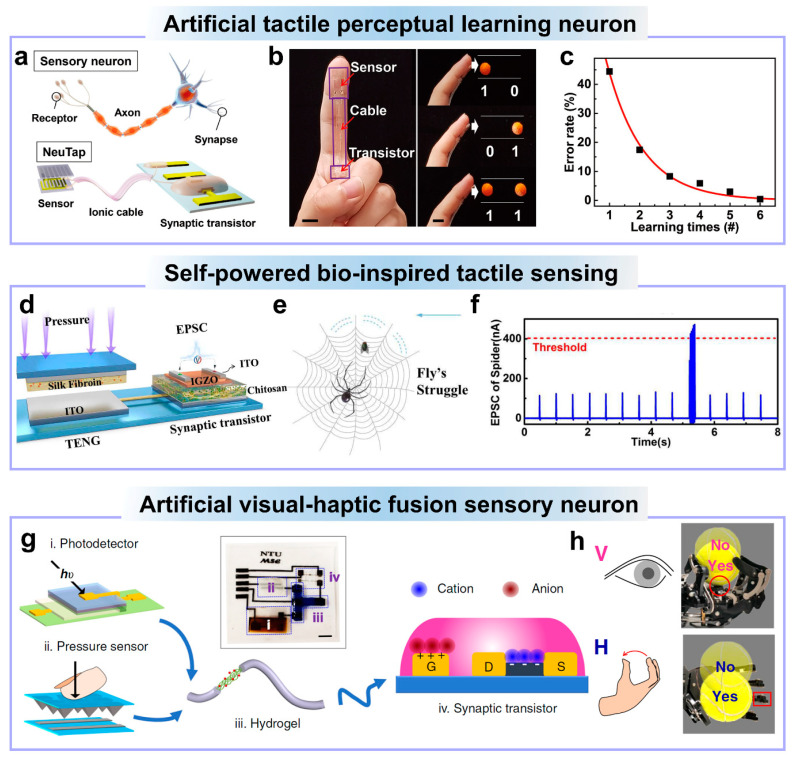
(**a**) Schematic illustration of a sensory neuron and an artificial sensory neuron based on a resistive pressure sensor, an ionic cable, and an oxide ionic transistor. (**b**) Image of the artificial sensory neuron on a finger and the pattern pairs and their corresponding two-bit code labels. (**c**) The tactile pattern classification error as a function of learning times. (**d**) Self-powered tactile sensing neuron, where TENG is used as the pressure sensor and powers the system and the ionic oxide transistor is used as synaptic processing unit. (**e**) Schematic illustration of a spider web. (**f**) The high-frequency fly struggle causes high-frequency vibration of the web, resulting in the large response of the oxide ionic synaptic transistor. (**g**) The oxide ionic transistor-based system for visual-haptic fusion. A photodetector and a resistive pressure sensor are used for visual and haptic perception, respectively. The ionic cable is used for signal transmission and fusion. The oxide ionic transistor is used for synaptic ionic processing. (**h**) The ‘YES’ or ‘NO’ position means visual or haptic feedback. (**a**–**c**) are reproduced from ref. [110]. Copyright 2018 WILEY-VCH Verlag GmbH & Co. KGaA, Weinheim. (**d**–**f**) are reproduced from ref. [109]. Copyright 2020 IEEE. (**g**,**h**) are reproduced from ref. [111]. Copyright 2020 the Author(s), a Creative Commons Attribution 4.0 International License.

Tactile perception, a fundamental aspect of human sensory experience, hinges upon a multifaceted sequence of activities encompassing sensing, refining, and learning [172]. As we touch and explore our surroundings, our sensory receptors gather data from various textures, temperatures, and pressures, which is then meticulously refined and processed by our neural networks, allowing us to discern nuances in texture, temperature, and shape. Moreover, the remarkable capability of our tactile system to adapt and learn over time further enriches our interactions. Wan et al. reported an artificial sensory neuron with tactile perceptual learning by integrating a resistive pressure sensor and an IWO ionic transistor [110]. As shown in Figure 7a, this tactile sensory system mainly includes a resistive tactile sensor, ionic cable, and synaptic transistor, which are used for sensing, information transmitting, and synaptic information processing, respectively. This artificial tactile sensory neuron is developed to carry out tactile pattern recognition. The experimental setup involved two distinct patterns positioned side by side in a single row, as shown in Figure 7b. To categorize and differentiate these pattern pairs effectively, a labeling scheme was established wherein the convex pattern within each pair was designated as “1”, while the flat pattern received a label of “0”. Consequently, each pattern pair acquired a binary code as its unique identifier, comprising the combinations “00”, “01”, “10”, and “11”, respectively. After training, the pattern recognition error can be reduced by 100 times after six times of learning, as shown as Figure 7c.

Triboelectric nanogenerators (TENGs) have garnered significant interest as self-powered pressure sensors due to their remarkable attributes, including exceptional sensitivity and rapid response to dynamic pressure changes [173]. Zhang et al. proposed a self-powered bio-inspired tactile sensory system [109]. A triboelectric nanogenerator is not only used as the tactile sensing part but also powers the system. An IGZO ionic transistor is used as the synaptic information processing unit, as shown in Figure 7d. The spider’s sensory organ exhibits an extraordinary degree of sensitivity to mechanical signals [174]. A notable example of this is observed in web-weaving spiders, which possess the remarkable ability to discern and accurately identify potential prey by detecting the subtle vibration signals transmitted through their own silk threads. Particularly, the vibration frequency of a cobweb induced by an insect’s struggle ranges from tens to hundreds of hertz, a distinct contrast to the low-frequency vibrations caused by wind, which is only a few hertz. Based on this fact, pressure pulses applied on the TENG at different frequencies are used as the vibration induced by an insect’s struggle or the wind. As shown is Figure 7f, high-frequency pressure pulses applied on the TENG can produce large post-synaptic response of the synaptic transistor reaching the threshold, while low-frequency compression leads to small post-synaptic response not reaching the threshold, mimicking the spider’s ability of identifying prey by sensing the vibration of cobwebs.

A human has five basic senses, namely touch, sight, hearing, smell, and taste. Our sensory organs transmit information to the brain to facilitate our comprehension and perception of the world. The environment around us is extremely sophisticated, full of various physical or chemical signal cues [175]. The human neuronal system efficiently processes various sensory cues to form an accurate representation of the environment. Wan et al. developed a visual–haptic fusion sensory neuron using a photodetector and a resistive pressure sensor connected to an oxide ionic synaptic transistor [111]. As shown in Figure 7g, the visual and haptic information cues are fused through hydrogel ionic coupling and then the fused signal is sent to the synaptic ionic transistor for further ionic/electronic processing. The visual–haptic fusion strategy can enhance robotic motion control and surpass unimodal methods, such as grabbing tennis balls. This signal fusion can advance the robotic design by improving sensory situational awareness.

## 7. Conclusions and Outlook

In this review article, we briefly introduce oxide ionic transistors for ionic dynamic neuromorphic computing. Firstly, the ionic process involving synaptic/neuronal information processing is introduced. Then, ionic dynamic synaptic/neuronal functions, especially spatiotemporal information processing, emulated by oxide ionic transistors are summarized. Later, artificial sensory neuron functions, where sensors are used for sensing and oxide electrolyte-gated transistors are used for ionic synaptic/neuronal processing, are introduced.

In addition to electrolyte transistors, several other neuromorphic computing technologies have been developed including CMOS integrated circuits, memristors, ferroelectric transistors, and floating-gate transistors. CMOS integrated circuits are the foundation of current computing devices. The shrinking of CMOS transistors via the last half a century via Moore’s law empowers the reduced energy consumption and operational frequency increase of CMOS circuits. However, the CMOS transistor was not specifically invented for neuromorphic computing, resulting in low efficiency for CMOS transistors to simulate a synapse or a neuron. It costs tens of CMOS transistors to simulate synapses or neurons. Memristors have a two-terminal structure, which makes it promising for ultra-high integration. After specialized neuron/synapse bionic design, volatile and non-volatile memristors are very promising for synaptic weight updating and neuronal integrate–fire functions, respectively. However, two-terminal memristors lack a select terminal, which may lead to select issues and unselected sneak path currents. The resistive coupling of the memristor-based circuit also makes it inferior compared with capacitive coupled transistors in static energy consumption. Compatibility with large-scale integrated circuits is a huge advance of ferroelectric and floating-gate transistors in neuromorphic computing. The multi-bit non-volatile memory performance of ferroelectric and floating-gate transistors makes them suitable for synaptic weight update. Compared with other technologies, electrolyte-gate transistors utilize the ions in the electrolyte to mimic synaptic/neuronal functions. In terms of working principle, it can more realistically emulate the neural functions because electrolyte-gated transistors and biological neural networks both employ ions as the information processing medium. Electrolyte transistors have an advantage in simulating neural ionic dynamics.

In order to further promote the practical application of the oxide ionic transistor in ionic dynamic neuromorphic computing, we here address the current challenges and offer recommendations along with potential research directions.

(1) Ionic neural functions extraction and refinement. Currently, some essential ionic neural functions like synaptic plasticity, spatiotemporal information processing, and sensory perception have been successfully implemented by various oxide ionic transistors. The human neural system has a highly interconnected complex structure and highly intelligent functions like pattern recognition and decision making. It contains about 100 billion neurons and even more highly interconnected synapses with them. The vast majority of current oxide ionic transistors have achieved biological intelligence at the device level. The implementation of more complex functions such as motion control and thinking is still in the early stages. Further research efforts are imperative to refine, abstract, and effectively implement more intricate neural functions. The advancement in complex neural function implementation requires collaborative efforts across multidisciplinary fields including neuroscience, materials, and electronics.
(2) Stability. Displays driven by oxide thin-film transistors have been used as mobile phone screens, which means that the stability problem of the IGZO material has been solved. Oxide ionic synaptic/neuronal transistors often involve an electrochemical doping or reaction process, which may cause some instability factors. Currently, a large variety of organic electrolytes are employed as the gate dielectric of oxide ionic neuromorphic transistors. The introduction of these organic compounds may cause some instability issues. Future research efforts could focus on encapsulation, which is an essential strategy for improving stability. By encapsulation, the devices are effectively shielded from environmental factors like oxygen, moisture, and mechanical stress. Moving forward, research efforts in this field should prioritize the exploration of highly stable organic/inorganic materials followed by encapsulation and continue to explore new ionic neuromorphic functions.
(3) Scalability. Device scaling means smaller footprint and lower power consumption. The channel length of oxide-based transistors is promising to scale below the 5 nm regime because of the unique wide bandgap and low dielectric constant. However, some of the oxide-based electrolyte synaptic/neuronal transistors adopt lateral-gate structure, as shown in Figure 2c. This lateral-gate structure provides the devices multiple inputs, which is very promising for neuronal information integration. But this lateral-gate structure will occupy much larger area and reduce integration. To achieve sustainable device scaling, manufacturing compatibility with existing fabrication processes is essential for future oxide-based electrolyte-gated transistors. Modern micro-nano electronic technology has achieved remarkable achievements worldwide. Neurons in the human brain are interconnected via synapses and arranged in a 3D manner, which is a great challenge for micro-nano technology. At present, the interconnection between massive neurons is not fully understood, which looks forward to the advancement of neuroscience. In order to realize the tremendous amount of interconnection, future research must focus on developing and optimizing 3D integration technology to meet neuromorphic interconnection requirements.
(4) Integration with existing systems. Integrating oxide electrolyte transistors with existing systems can offer a range of advances in the fields of bioelectronics, neuromorphic computing, and flexible electronics. Due to the compatibility of oxide electrolyte transistors with an ionic aqueous environment, it is possible for oxide electrolyte transistors to interface with biological systems. Oxide electrolyte transistors have been proved to be efficient in mimicking synaptic/neuronal ionic computing. When integrated with current CMOS circuits, they will combine the advantages of both, that is, the powerful digital signal processing capabilities of CMOS circuits and the efficient bionic capabilities of synaptic/neuron functions of oxide electrolyte transistors. The biggest challenge in integrating oxide-based electrolyte transistors and existing CMOS circuits comes from their compatibility. Most organic electrolytes are not compatible with the CMOS process. To realize the integration with current existing systems, future efforts require more on the development of micro-nano process- compatible inorganic electrolyte transistors.
(5) Power consumption. The power consumption of biological systems is estimated to be a few tens of pico-joules per event [176]. Because of the high capacitance, the working voltage of oxide-based electrolyte-gated transistors can be reduced to less than 2.0 V, and the energy consumption of oxide-based electrolyte synaptic transistors can be reduced to levels comparable to that of biological synapses. The low-off current of oxide transistors ensures low static power consumption. Nevertheless, the overall system power consumption including peripheral circuits and oxide-based electrolyte transistor neural simulation core is believed to be much larger than that of biological systems. In order to further reduce power consumption, other working regimes can be explored like subthreshold operation mode.

The future work in the field of oxide-based electrolyte-gated transistors for neuromorphic computing can focus on the following: (1) Material optimization. Optimize the oxide-based channel material and electrolyte material and make it more stable and compatible with CMOS processes. (2) Device design. Investigating more feasible device design to enhance the ionic dynamic function implementation and efficiency of oxide-based electrolyte-gated transistors for neuromorphic computing. (3) Integration with other technologies. Integrating oxide-based electrolyte-gated transistors with other technologies like CMOS circuits, memristors, or floating-gate transistors can combine the advantages of both technologies and realize more powerful artificial neural networks. The potential applications of oxide-based electrolyte transistors for neuromorphic computing include bio-inspired sensing and prosthesis, edge computing, and brain–machine interfaces.
